# The Mediating Role of Body Acceptance in Explaining the Relation of Mindfulness, Self-Compassion and Mindful Eating to Body Image in Gay Men and Bisexual Men

**DOI:** 10.1007/s12671-023-02095-7

**Published:** 2023-03-09

**Authors:** Harvey Regan, Rebecca Keyte, Michael Mantzios, Helen Egan

**Affiliations:** Department of Psychology, Faculty of Business, Law and Social Sciences, Birmingham City University, Room C325, The Curzon Building, 4 Cardigan St, Birmingham, B4 7BD United Kingdom

**Keywords:** Self-compassion, Body-acceptance, Gay men, Mindfulness, Body image

## Abstract

**Objectives:**

Mindfulness and mindfulness-based constructs, such as self-compassion and mindful eating, have been positively associated with healthier eating and body related perceptions. Exploration of mindfulness and related concepts have not been investigated extensively in gay and bisexual men, a population where eating and body related concerns have been found to be widespread.

**Method:**

Participants completed an online questionnaire, assessing mindfulness, self-compassion, mindful eating, body image and body acceptance. Correlation analysis and further mediation analysis was conducted to explore the relations between these constructs within the present sample (*n* = 163).

**Results:**

A community sample showed a positive association of body image to mindfulness-based concepts, and negative to body non-acceptance, within the target population. Mediation analysis showed the role of body acceptance in explaining the relation between mindfulness, self-compassion and mindful eating to body image.

**Conclusions:**

Findings highlight the importance of body acceptance when considering the development of a mindfulness or compassion-based intervention to attenuate body related issues among gay and bisexual men.

**Preregistration:**

This manuscript has not been preregistered.

Mindfulness and mindfulness-based concepts, such as self-compassion and mindful eating, have been utilised in research throughout health psychology, especially in assisting populations engaging with problematic eating behaviours and experiencing body image concerns (Jordan et al., 2014; Mantzios, & Wilson, 2015; Tihanyi et al., 2016; Wasylkiw et al., 2012). Mindfulness has been described as paying attention to the present moment, with a non-judgemental attitude (Kabat-Zinn, 2015). Exploration of mindfulness has promoted the development and efficacy of mindfulness (Balciuniene et al., 2021) and compassion-based interventions (Albertson et al., 2015) aiming to attenuate body image and eating related issues in the general population, but also in more specific populations where these issues are prevalent (e.g., adolescent females; individuals with a diagnosis of Cystic Fibrosis) (Egan et al., 2021; Hussein et al., 2017). Exploration of mindfulness and related concepts have not been investigated extensively in gay and bisexual men although evidence shows that this is a population who are also at higher risk of developing eating and body image related issues (e.g., Fussner & Smith, 2015; McClain & Peebles, 2016; Tran et al., 2020). The development of interventions which consider the specific experiences of different populations increases the efficacy of such interventions, hence the need for increased knowledge of experiences of body issues in gay and bisexual men.

Within Western culture, presentation of “thin” or “slim” female body types have contributed to poor body image perception among younger female populations (Bombak et al., 2019). Those who do not meet this ideal expectation often display lower body image and dissatisfaction (Bombak et al., 2019) and perceive a lack of acceptance of their body shape from peers, family, and friends (Tylka & Homan, 2015). Body acceptance has been defined as an acknowledgement of feeling unsatisfied with some aspects of the body but accepting these non-judgementally (Tylka & Wood-Barcalow, 2015); it is often aligned with “fat acceptance” though there are many facets to body acceptance amongst different populations (Ruggiero et al., 2000). The perceived “body acceptance by others” (Swami et al., 2020) in relation to those who are close to the individual (*family and friends*) has also been linked to body image and mental health outcomes (Layman et al., 2021). Evidence shows that higher rates of body acceptance relate to positive body image (Swami et al., 2021). A higher prevalence for body related issues has been found in female populations including a lack of body acceptance, leading to poor body image and body dissatisfaction (Santonastaso, 1995). A large collection of research has attributed this to women internalizing the “thin” body ideals portrayed by the media (Pidgeon & Appleby, 2014). While findings generally indicate a thin “ideal body” proposition, further explorations of body acceptance and how this relates to body image and mindfulness constructs is needed.

Research that explores body image in men does not often depict the sexuality of participants and therefore does not consider the specific experiences of gay and bisexual men (Fussner & Smith, 2015). This omission is important, as recent literature has highlighted gay and bisexual men as experiencing a higher prevalence of eating and body related issues when compared with heterosexual men (Blashill, 2010). Brewster et al. (2017) suggest that the pressure upon gay men to conform to the high standards of bodily appearance in the gay community could result in disordered eating and body dissatisfaction. Yelland and Tiggemann (2003) compared measures assessing disordered eating and desire for muscularity in gay men, using heterosexual men and women as control groups. Gay men scored higher in the disordered eating and desire for muscularity measures than both the control groups (Yelland & Tiggemann, 2003). This suggests that the aspirational male gay body type is both lean and muscular. This body ideal can be difficult to achieve and maintain and may help to explain the higher rates of disordered eating. A perceived failure to achieve such body ideals may also negatively affect wellbeing in gay men (Brewster et al., 2017; Regan et al., 2021; Yelland & Tiggemann, 2003).

Qualitative explorations of body ideals in relation to social perceptions and the impact on the individual have reflected similar findings (Morgan, & Arcelus, 2009). Regan et al. (2021) highlighted the appearance-based judgement experienced by gay men who visited “gay spaces”, and how this related to a lack of body acceptance within gay men. Further findings showed that participants were judgemental of themselves when they had eaten unhealthy food, particularly when consumption was unplanned or eaten without attention (i.e., mindless eating). This research highlights the lack of body acceptance experienced and the perceived importance of attaining or maintaining a slim or muscular body type to be “accepted” within this community (Regan et al., 2021). The potential of mindfulness and self-compassion to improve body acceptance and body image is proposed (Brewster et al., 2017; Regan et al., 2021; Yelland & Tiggemann, 2003).

Literature presents several explorations of mindfulness-based interventions and their effectiveness at attenuating body related issues within the general and more specific populations. Balciuniene et al. (2021) tested an 8-week intervention programme utilising mindfulness based physical exercise and educational sessions with a sample of female college students who showed an increase in body image scores post intervention. Similarly, Zamzami et al. (2015) demonstrated that mindfulness-based exercise attenuated lower body image in female students. This provides evidence of the effectiveness of mindfulness-based interventions when addressing body related issues in specific populations (Balciuniene et al., 2021; Zamzami et al., 2015).

Self-compassion is a mindfulness-based construct which describes compassion directed towards oneself, comprising of three main elements, kindness, a sense of common humanity and mindfulness (Germer & Neff, 2013). This concept has been explored within body image and eating literature, where higher levels of self-compassion relating to higher levels of body satisfaction and lower reports of disordered or maladaptive eating (Egan et al., 2018; Mantzios & Egan, 2017; Rahimi-Ardabili et al., 2018; Regan et al., 2021). Wasylkiw et al. (2012) explored self-compassion and self-kindness in relation to body image in a sample of female university students. Higher levels of self-compassion and self-kindness were found to be predictors of higher levels of body image. Self-compassion-based interventions have also shown efficacy in reducing body dissatisfaction in female populations, Albertson et al. (2015) tested a daily audio guided self-compassion based-meditation intervention over 3 weeks, results showed higher levels of self-compassion which were associated with higher levels of body image.

Emerging research has looked at the potential influence mindful eating may have on reducing not only maladaptive eating behaviours, but also body dissatisfaction (Olvera-Ruvalcaba & Gómez-Peresmitré, 2021). Mindful eating encompasses non-judgment, eating with awareness and engaging with the physical and emotional sensations associated with eating (Mantzios, 2021). Ponde Nejadan et al. (2018) explored mindful eating, body image and quality of life in a sample of married Iranian women, showing increases of mindful eating related to increases in body image and quality of life. This research suggests that mindful eating may also play a role in promoting positive body image. Further links between mindful eating and body image are presented by Webb et al. (2018) who investigated the impact of family talk around mealtimes. Results showed that self-denigrating talk was inversely linked with mindful eating while increases in mindful eating behaviours increased positive body image and appreciation. Research proposes the importance of mindful eating (Mantzios et al., 2018), and by extension, of mindful-eating based interventions (Hussain et al., 2017; Mantzios et al., 2020a, b), when considering its potential impact on positive body image.

The links between body image and mindfulness-based concepts have been discussed (Jordan et al., 2014; Tihanyi et al., 2016; Wasylkiw et al., 2012); however, the potential link of body acceptance to mindfulness has not been extensively explored. Exploration of these concepts within gay and bisexual men would highlight elements that may be important when considering a mindful or compassion-based intervention to attenuate body and eating related issues in gay and bisexual men. The aim of this study is to explore body image and the potential relationships to mindfulness, self-compassion, and mindful eating in gay and bisexual men to inform future mindful and/or compassion-based intervention to attenuate body related issues. Importantly, the present research assumes the close association of body acceptance to body-image, and the congruent nature of acceptance to mindfulness, self-compassion, and mindful eating to be a significant indicator of promoting healthier changes towards body perceptions.

## Method

### Participants

All participants (*n* = 163, *M*_*age*_ = 37.29, *SD* = 12.07; *M*_*BMI =*_ 26.37, SD = 4.94) were English-speaking, from the UK and self-identified as either *gay* (89%, *n* = 145), bi-sexual (7.4%, *n* = 12), or heteroflexible (1.2%, *n* = 2) with 2.4 % (*n* = 4) not disclosing any information regarding sexuality. Eligibility criteria included individuals who were over the age of 18 years old and those who has not received a diagnosis of an eating or body-related disorder within the past 2 years, this was screened for within the Participant information sheet and the Informed consent form. According to Fritz and MacKinnon (2007), a sample size of 163 participants would enable observations of an indirect effect of a medium-sized alpha pathway coefficient (i.e., *predictor to mediator*) and a medium-sized beta pathway coefficient (i.e., *mediator to criterion*) at 80% power using bias-corrected bootstrapping estimating procedures (Table 1).

**Table 1:** Participant demographic information

Variable	Participants (*n* = 163)
Sexuality	
Gay	145
Bisexual	12
Heteroflexible	2
Non-disclosure	4
Gender	
Trans-male	1
Non-binary	1
Cis male	130
Gender fluid	4
Gender non-conforming	5
Non-disclosure	22
Ethnicity	
White British	115
White Irish	5
White and black Caribbean	4
African	3
Caribbean	4
White and Black African	2
South Asian	6
Non-disclosure	24

### Procedure

Participants were recruited through volunteer sampling; an advert for the study outlining its nature, target population and link to the questionnaire was used for recruitment. This poster was disseminated by the research team through social media platforms, highlighting the study information and linking to the questionnaire platform to potential participants. The online survey platform Qualtrics was used to contain the questionnaire. Upon clicking the link, participants were presented with an online version of the Information sheet and Consent form which had to be viewed and agreed to before the questionnaire could be accessed. Once all measures were completed, participants were presented with the Debrief form. This included information regarding the contact details of the researcher, further support, and details of their right to withdraw their data from the study should they wish to do so at a later date. Data were collected from March until August 2021. Ethical approval was received from The Business Law and Social Sciences Ethics Committee at Birmingham City University (Regan /#7972 /sub3 /R(B) /2021 /Jan /BLSS FAEC).

### Measures

*Participant information sheet*. Participants were asked to report their age, gender, height, weight, ethnicity, smoking and exercise engagement.

The *Sussex-Oxford Compassion for the Self* (SOCS-S; Gu et al., 2020) is a 20-item scale containing 5 sub-scales (*Recognising suffering; Understanding the universality of suffering; Feeling for the person suffering; Tolerating uncomfortable feelings; Acting or being motivated to act to alleviate suffering*). Total scores were calculated and used within the analysis; with the higher the score meaning higher levels of self-compassion. Responses were recorded using a 5-point Likert scale (1 = *Not at all true*, 2 = *Rarely true*, 3 = *Sometimes true*, 4 = *Often true*, 5 = *Always true*), sample items include: “I notice when I’m feeling distressed” and “I connect with my own suffering without judging myself”. Cronbach’s alpha and McDonald’s omega were used to assess the scale reliability for the SOCS-S in the present research (α = 0.95, ω = 0.95).

The *Body Image Acceptance and Action Questionnaire* -5 (BI-AAQ-5; Basarkod, Sahdra & Ciarrochi, 2018) is a short form of the *Body image – Acceptance and Action Questionnaire* (BI-AAQ-5) which aims to assess body image acceptance. Total scores were calculated and used within the analysis; with a higher score meaning lower levels of body-acceptance (*or higher levels of body non-acceptance*). The BI-AAQ-5 is a 5-item scale where responses are recorded using a 7-point Likert scale (1 = *Always true* and 7 = *Never true*). Sample items include: *“*Worrying about my weight makes it difficult for me to live a life that I value” and “I shut down when I feel bad about my body shape or weight”. Cronbach’s alpha and McDonald’s omega were used to assess the scale reliability for the BI-AAQ in the present research (α = 0.92, ω = 0.92).

The *Dresden Body Image Questionnaire* (DBIQ; Scheffers et al., 2017) is a 35-item questionnaire with positively and negatively worded statements comprising of five subscales (*Body Acceptance, Vitality, Physical Contact, Sexual Fulfilment and Self-aggrandizement*). The DBIQ aims to assess body image, with higher scores meaning higher levels of a more positive perception of body image; total scores were calculated and used within the analysis. Responses were recorded using a 5-point Likert scale (1 = *Not at all true*, 2 = *Rarely true*, 3 = *Sometimes true*, 4 = *Often true*, 5 = *Always true*), sample items include: “I wish I had a different body” and “I use my body to attract attention”. Cronbach’s alpha and McDonald’s omega were used to assess the scale reliability for the BDIQ in the present research (α = 0.91, ω = 0.91).

The *Mindful Eating Behaviour Scale* (MEBS; Winkens et al., 2018) is a 20-item scale, and has 5 subscales (*Focused Eating, Eating with Awareness, Eating without Distraction, Hunger and Satiety Cues*). Total scores were calculated and used within the analysis; with a higher score meaning higher levels of mindful eating. Responses were recorded using a 4-point Likert scale (1 = *Never* to 4 = *Usually*), sample items include: “I wish I could control my eating more easily” and “I trust my body to tell me when to eat”. Cronbach’s alpha and McDonald’s omega were used to assess the scale reliability for the MEBS in the present research (α = 0.80, ω = 1.08).

*The Five Facet Mindfulness Questionnaire* (FFMQ-15; Gu et al., 2016) is a 15-item scale, and comprises of 5 subscales (*Observing items, Describe items, Acting with awareness items, Non-judging items, Non-reactivity items*). Total scores were calculated and used within the analysis; with the higher the score meaning higher levels of mindfulness. Responses were recorded using a 5-point Likert scale (1 = *Never or very rarely true* to 5 = *Very often or always true*), sample items include: “I’m good at finding words to describe my feelings” and “I find myself doing things without paying attention”. Cronbach’s alpha for the FFMQ in the present research was *α =* 0.67. McDonald’s omega was used to assess the scale reliability for the FFMQ in the present research, but the low association of the items and the proposed poor model fit did not allow for a score until Observe items (i.e., 1, 6, and 11) and Item 5 (non-reactivity) were removed (ω = 0.62).

### Data Analyses

All statistical analyses were conducted using IBM SPSS 25. A total of 44 participants were excluded from the study due to incomplete or missing data, which took place within the initial screening process, leaving a total of 163 participants completing all measures described within this study. A significance value of <0.05 was used to determine significant relationships between variables. Bivariate correlation analysis was used to determine the relationship between variables explored within the questionnaire. Mediation analyses were conducted using Hayes’ (2017) PROCESS macro (Model 4) with a bootstrap sample of 5000. Confidence intervals (CI) do not cross zero and are considered significant when upper and lower boundaries are corrected to 95%. Body acceptance was used as a mediator to explore the effect on the relationship between Mindfulness, Self-compassion, and Mindful Eating on Body Image.

## Results

### Correlation Analyses

Pearson’s Bivariate correlation coefficient was employed using significant values between variables (Body acceptance, Body image, Mindfulness, Self-compassion, and Mindful eating), as well as means and standard deviations as presented in Table 2. Significant negative associations were observed between body non-acceptance and body image (*r* = -0.63, *p* < 0.001), suggesting that with higher body image there is a decrease of non-body acceptance (*essentially meaning the higher body image, the higher the scores on measures assessing body acceptance*). Significant negative associations were observed between body non-acceptance, mindfulness (*r* = -0.42, *p* < 0.001), self-compassion (*r* = -0.50, *p* < 0.001) and mindful eating (*r* = -0.43, *p* < 0.001). The higher the body non-acceptance, the lower the scores in mindfulness, self-compassion and mindful eating (*essentially meaning the higher body acceptance the higher scores in mindfulness, self-compassion and mindful eating*). Significant positive associations were observed between body image, mindfulness (*r* = 0.32, *p* < 0.001), self-compassion (*r* = 0.50, *p* < 0.001) and mindful eating (*r* = 0.48, *p* < 0.001). As body image increased, scores on measures assessing mindfulness, self-compassion, and mindful eating also increased.

**Table 2:** Means and standard deviations of variables, and bivariate correlations between body image, body acceptance, mindfulness, self-compassion and mindful eating

Scales	1	2	3	4	5	*M*	*SD*
(1) BIAAQ						18.82	8.21
(2) DBIQ	-0.63^**^					106.75	20.25
(3) FFMQ	-0.41^**^	0.32^**^				46.26	7.29
(4) SOCS-S	-0.50^**^	0.50^**^	0.65^**^			66.69	14.71
(5) MEBS	-0.43^**^	0.48^**^	0.41^**^	0.52^**^		67.85	10.01

*BI-AAQ-5 – Body image Acceptance and Action scale (higher scores represent higher body non-acceptance); DBIQ – Dresden Body Image Questionnaire; FFMQ – Five Facet Mindfulness Questionnaire; SOCS-O - The Sussex-Oxford Compassion for Others; MEBS – Mindful Eating Behaviour Questionnaire.*

** Correlation is significant at the 0.01 level (two-tailed).

### Mediation Analyses

**Fig. 1:**
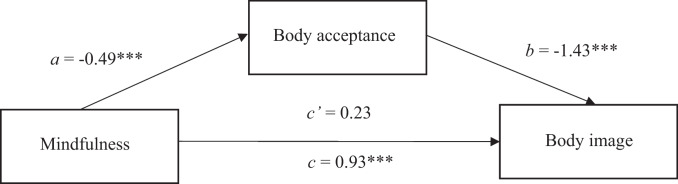
The mediating effect of body acceptance in the relationship between mindfulness and body image. Note: All presented effects are unstandardised; *a* is the effect of Mindfulness on body acceptance; *b* is the effect of body acceptance on body image; *c*’ is the direct effect of mindfulness on body image; *c* is the total effect of mindfulness on body image. * *p < *0.05, ** *p < *0.01, *** *p < *0.001. *Further note: B-IAAQ-5 – Body image Acceptance and Action scale (higher scores represent higher body non-acceptance)*

Further analysis explored the mediating effect of body acceptance on the relationships of mindfulness, self-compassion and mindful eating to body image. First, mindfulness was entered as the predictor variable and body image was entered as the outcome variable. Body acceptance was entered as the potential mediating variable. Findings indicated that mindfulness indirectly relate to body image, through its relationship with body acceptance. Mindfulness significantly predicted body acceptance (*b* = -0.49, *t* = 5.47, *p* < 0.001), as scores on mindfulness increased, scores on body acceptance decreased which related to body acceptance significantly predicting body image (*b* = 1.43, *t* = 7.77, *p* < 0.001). A 95% bias-corrected confidence interval based on 5000 bootstrap samples indicated that there was an indirect effect (*b* = 0.70) which was above zero (*CI* = 0.41, 1.00) Fig. 1.

**Fig. 2:**
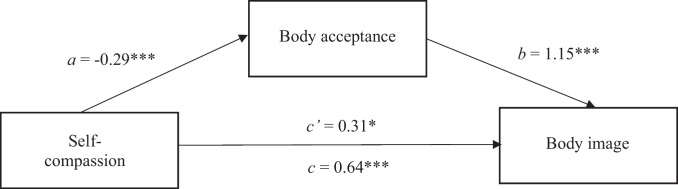
The mediating effect of body acceptance in the relationship between self-compassion and body image. Note: All presented effects are unstandardised; a is the effect of self-compassion on body acceptance; b is the effect of body acceptacnce on body image; c’ is the direct effect of self-compassion on body image; c is the total effect of self-comapssion on body image. * p < 0.05, ** p < 0.01, *** p < 0.001*. Further note: BI-AAQ-5 – Body image Acceptance and Action scale (higher scores represent higher body non-acceptance)*

Secondly, self-compassion was entered as the predictor variable, body image as the outcome variable and body acceptance as the potential mediator. Findings indicated that self-compassion indirectly related to body image, through its relationship with body acceptance. Self-compassion significantly predicted body acceptance (*b* = -0.29, *t* = 5.41, *p* < 0.001), as scores of self-compassion increased, scores on body acceptance decreased which related to body acceptance significantly predicting body image (*b* = 1.15, *t* = 6.60, *p* < 0.001). A 95% bias-corrected confidence interval based on 5000 bootstrap samples indicated that the indirect effect (*b* = 0.33) was above zero (*CI* = 0.18, 0.54) Fig. 2.

**Fig. 3:**
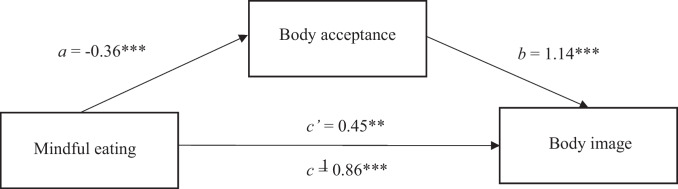
The mediating effect of body acceptance in the relationship between mindful eating and body image. Note: All presented effects are unstandardised; a is the effect of mindful eating on body acceptance; b is the effect of body acceptacnce on body image; c’ is the direct effect of mindful eating on body image; c is the total effect of mindful eating on body image. * p < 0.05, ** p < 0.01, *** p < 0.001*. Further note: BI-AAQ-5 – Body image Acceptance and Action scale (higher scores represent higher body non-acceptance)*

Lastly, mindful eating was entered as the predictor variable, body image as the outcome variable and body acceptance as the potential mediator. Findings indicated that mindful eating indirectly related to body image, through its relationship with body acceptance. Mindful eating significantly predicted body acceptance (*b* = -0.36, *t* = 5.30, *p* < 0.001), as scores of mindful eating increased, scores on body acceptance decreased which related to body acceptance significantly predicting body image (*b* = 1.14, *t* = 5.70, *p* < 0.001). A 95% bias-corrected confidence interval based on 5000 bootstrap samples indicated that the indirect effect (*b* = -0.42) was above zero (*CI* = 0.23, 0.62) Fig. 3.

## Discussion

The aim of this research was to explore body image, body acceptance and their relationship to mindfulness, self-compassion, and mindful eating among gay and bisexual men. Exploring these concepts among the current population provides novel insight into body image and their relationships to mindfulness and related concepts (i.e., self-compassion and mindful eating) among a previously underrepresented community. Furthermore, the potential relationship between mindfulness, mindful eating and self-compassion to body-image, and the potential of explaining such relationships through body acceptance was also explored. Findings indicated that body acceptance mediated the relationship between mindfulness, self-compassion and body image, and mindful eating and body image. This corresponds with previous literature that outlines the link between mindfulness (Zamzami et al., 2015), self-compassion (Mantzios & Egan, 2017, 2018) and mindful eating (Ponde Nejadan et al., 2018) to body image. These findings presented within a gay population reflect the outcomes of research within general and more specific populations (Albertson et al., 2015). This research also shows the potential association of body acceptance to mindfulness and body image within this population. The indication of the relationships between mindfulness, self-compassion and mindful eating to body image can be explained through body acceptance, highlighting its importance within this community.

Correlation analysis also provided some novel and interesting findings. Within this population, body image related positively to self-compassion, mindfulness and mindful eating. Research exploring mindfulness-based concepts and body image in female populations are similar to the current findings presented (Balciuniene et al., 2021; Zamzami et al., 2015); suggesting that an increase in body image relates to a more mindful and compassion view of the self. Higher body acceptance also related positively to mindfulness, self-compassion and mindful eating, suggesting that this construct relates similarly to body image in its relationship to mindfulness-based constructs.

It is important to consider the demographic of participants included within this sample. The average BMI of participants (26.37) falls into the category deemed as “overweight”; meaning the conclusions drawn from this sample can only be attributed to an overweight population. The majority of participants also identified themselves as “White British” (*n* = 115). Future research should endeavour to capture the experiences of queer people of colour within their research, helping to provide insight into body- and mindfulness- related constructs among diverse samples.

The findings from the present study should inform future research and practice in aiming to attenuate body related issues in this population. The role of body acceptance here also provides a clear link that this construct is related to mindfulness, self-compassion, mindful-eating, and body image. Future research exploring these concepts or investigating the efficacy of interventions should consider this construct in relation to body image.

### Limitations and Future Research Directions

This research concedes the following limitations which are significant to consider for future research. First, the cross-sectional nature of this study does provide limited insight, and qualitative explorations should gather more in-depth data from gay men. Second, conclusions can only be drawn from the period that data was collected. Consideration should be given to data being collected during the COVID-19 pandemic (March to August 2021) where variations of restrictions were in place across the United Kingdom. This could have played some role in altering participants’ perceptions of body image and body acceptance, specifically in relation to mindfulness-based constructs. This could be specifically prominent within this community; whereby the social influence of perceived body ideals is compounded by perceptions of other gay men, particularly when in ‘gay spaces’ which negatively impacts on body self-acceptance (Regan et al., 2021). Future research should explore these concepts within gay and bisexual men in more standard social parameters to gain a more a comprehensive understanding.

All scales and corresponding items included in this study were completed at one time by participants, the anticipated completion time was around 15-20 minutes. It is also important to consider the potential risk of survey fatigue experienced by participants in completing a questionnaire with many items and the implications this may have had on the results. Common methods bias (or variance) is a well-documented phenomenon observed in research based on self-reported measures. Multiple constructs are measured on multiple-item scales presented within the same questionnaire which can lead to spurious effects due to the measurement instruments rather than to the constructs being measured. For example, participants are asked to report their own perceptions on two or more constructs in the same questionnaire; this is likely to produce spurious correlations among the items measuring these constructs owing to response styles, social desirability, priming effects which are independent from the true relationships presented among the constructs being measured (Podsakoff et al., 2012). It is also important to consider the impact of the reliability score (Cronbach Alpha) for the FFMQ, as this was below the widely accepted 0.70 (0.67).

The disproportionate number of gay men who took part in this study when compared to bisexual men, highlights a lack of balance when considering the conclusions drawn from this research. The office for National Statistics stated that in 2019, the percentage of gay and bisexual men within the UK was 1.9% (*gay men*) and 0.6% (*bisexual men*) (Sanders, 2020)*;* meaning the data from this research does not reflect the representation of bisexual men within the wider population. Future research should endeavour to include a more diverse sample, to produce a balanced approach to drawing conclusions around the body image and related concepts of gay and bisexual men. The authors also consider the complexity of defining “gay men” or “bisexual men”. Non-binary, non-conforming and gender fluid individuals were included within this sample, the authors fully recognise that these individuals may or may not be comfortable with the label “men”. The inclusion of gender minorities within this sample are to strive to provide a greater inclusion of queer experiences within research, and not to label or make assumptions about participants’ gender.

Further research is needed to develop the understanding of mindfulness and related concepts within this community to aid in the development of an appropriate intervention. The necessity for a suitable intervention to attenuate eating and body related issues experienced by gay and bisexual men is clear. Mindful and compassion-based interventions have been effective in reducing body related issues within other populations (Balciuniene et al., 2021; Zamzami et al., 2015), therefore, evaluating the efficacy of such interventions within the gay population may provide novel research. This research shows the unique role of body acceptance to mindfulness, self-compassion and mindful eating when relating these concepts to body image. This provides insight into the potential addition of body acceptance and mindfulness-based concepts when considering potential avenues in overcoming body-related issues experienced by gay and bisexual men.

## Data Availability

Data available from the corresponding author upon request.
